# *Ganoderma pfeifferi* Bres. and *Ganoderma resinaceum* Boud. as Potential Therapeutic Agents: A Comparative Study on Antiproliferative and Lipid-Lowering Properties

**DOI:** 10.3390/jof10070501

**Published:** 2024-07-19

**Authors:** Milena Rašeta, Marko Kebert, Jovana Mišković, Saša Kostić, Sonja Kaišarević, Nebojša Stilinović, Saša Vukmirović, Maja Karaman

**Affiliations:** 1 Department of Chemistry, Biochemistry and Environmental Protection, Faculty of Sciences, University of Novi Sad, Trg Dositeja Obradovića 3, 21000 Novi Sad, Serbia; 2 ProFungi Laboratory, Department of Biology and Ecology, Faculty of Sciences, University of Novi Sad, Trg Dositeja Obradovića 2, 21000 Novi Sad, Serbia; jovana.maric@dbe.uns.ac.rs (J.M.); maja.karaman@dbe.uns.ac.rs (M.K.); 3 Institute of Lowland Forestry and Environment, University of Novi Sad, Antona Čehova 13d, 21000 Novi Sad, Serbia; kebertmarko@gmail.com (M.K.); sasa.kostic@uns.ac.rs (S.K.); 4 Department of Biology and Ecology, Faculty of Sciences, University of Novi Sad, Trg Dositeja Obradovića 2, 21000 Novi Sad, Serbia; sonja.kaisarevic@dbe.uns.ac.rs; 5 Department of Pharmacology, Toxicology and Clinical Pharmacology, Faculty of Medicine, University of Novi Sad, Hajduk Veljkova 3, 21000 Novi Sad, Serbia; nebojsa.stilinovic@gmail.com (N.S.); sasavukmirovic99@gmail.com (S.V.)

**Keywords:** *Ganoderma*, natural products, health benefits, fungal extracts, anticancer activity, liver protection, kidney protection, mineral composition

## Abstract

Medicinal mushrooms, especially *Ganoderma* species, hold immense promise for the production of a wide range of bioactive compounds with various effects. The biochemical potential of indigenous fungal strains, specific to a region, could play a critical role in the continuous search for novel strains with superior activities on a global scale. This research focused on the ethanolic (EtOH) and hot-water (H_2_O) extracts of fruiting bodies of two wild-growing *Ganoderma* species: *G. pfeifferi* and *G. resinaceum,* with the aim of assessing their nutritional (total carbohydrate content-TCC) and mineral composition in relation to bioactive properties: antioxidant, antiproliferative and lipid-lowering. Atomic absorption spectrophotometry (AAS) revealed that *G. pfeifferi* is a promising source of minerals that are essential for numerous physiological functions in the human body like bone health and muscle and nerve function, with Ca (4.55 ± 0.41 mg/g d.w.) and Mg (1.33 ± 0.09 mg/g d.w.) being the most abundant macroelement present. Zn, Mn, and Cr were particularly notable, with concentrations ranging from 21.49 to 41.70 mg/kg d.w. The EtOH extract of *G. pfeifferi* demonstrated significantly elevated levels of TCC, essential macromolecules for energy and structural functions in the body, with higher quantities of all three standard carbohydrates detected in this type of extract. Similar to the revealed composition, the same species, *G. pfeifferi,* stood out as the most prominent antioxidant agent, with the H_2_O extract being stronger than EtOH in the ABTS assay (86.85 ± 0.67 mg TE/g d.w.), while the EtOH extract displayed the highest anti-OH^•^ scavenging ability (IC_50_ = 0.18 ± 0.05 μg/mL) as well as the most notable reducing potential among all. The highest antiproliferative effect against the breast cancer cell line (MCF-7), were demonstrated by the H_2_O extracts from *G. resinaceum* with the most pronounced activity after 24 h (IC_50_ = 4.88 ± 0.50 μg/mL), which surpasses that of the standard compound, ellagic acid (IC_50_ = 33.94 ± 3.69 μg/mL). Administration of both *Ganoderma* extracts mitigated diabetic lipid disturbances and exhibited potential renal and hepatic protection in vivo on white Wistar rats by the preservation of kidney function parameters in *G. resinaceum* H_2_O pre-treatment (urea: 6.27 ± 0.64 mmol/L, creatinine: 50.00 ± 6.45 mmol/L) and the reduction in ALT levels (17.83 ± 3.25 U/L) compared to diabetic control groups treated with saline (urea: 46.98 ± 6.01 mmol/L, creatinine: 289.25 ± 73.87 mmol/L, and ALT: 60.17 ± 9.64 U/L). These results suggest that pre-treatment with *G. resinaceum* H_2_O extracts may have potential antidiabetic properties. In summary, detected microelements are vital for maintaining overall health, supporting metabolic processes, and protecting against various chronic diseases. Further research and dietary assessments could help determine the full potential and applications of the two underexplored *Ganoderma* species native to Serbia in nutrition and health supplements.

## 1. Introduction

The integration of traditional knowledge with modern scientific research continues to uncover valuable natural compounds with potential health benefits. The rising incidence of chronic diseases due to aging and lifestyle changes underscores the need for effective treatments [1,2]. Fungi, with their rich nutritional profile and therapeutic properties, offer promising avenues for addressing global health concerns, particularly oxidative stress. Leveraging ancient folk remedies, many of which form the basis of modern medications, can enhance our approach to managing and preventing these prevalent health issues [3]. For instance, recent research has focused on natural products for the prevention and treatment of metabolic syndrome, with particular attention being paid to medicinal mushrooms [4,5]. Among these, the *Ganoderma* species, known for their extensive use in traditional medicine, have shown promising potential [6,7]. *Ganoderma* species, such as *G. resinaceum*, have been documented to possess various bioactive compounds, including nortriterpenoids (lucidone A–F, H, I–K, ganosineniol B–C, ganoderense F, and ganosineniol), which exhibit a wide range of beneficial biological activities [8]. These activities include anti-inflammatory, antioxidant, and antidiabetic effects, which are particularly relevant to combating the multifaceted aspects of metabolic syndrome [6,8].

Species belonging to the genus *Ganoderma* (P. Karst.) have been utilized as a natural resource in Traditional Chinese Medicine (TCM) for over two hundred years [3,9]. Nowadays, they have been extensively studied for their various health benefits [6,7,10,11,12,13,14,15]. In 1889, Patouillard expanded the genus to include 48 species, while data from 2022 indicate that the genus now comprises 181 species [16]. Among them, *G. lucidum* is globally recognized, owing to its use in TCM as the “Mushroom of Immortality” [17]. Utilization of *Ganoderma* spp. has primarily relied on observations and accounts of its efficacy in cancer prevention and treatment, combating infections, modulating the immune system, and regulating blood pressure, among other medicinal benefits [6,7]. The medicinal properties of *Ganoderma* spp. primarily stem from the abundance of various bioactive compounds they produce, notably triterpenoids like ganoderic acids, which have been reported to exhibit anti-hypercholesterolemic, anticancer, hepatoprotective, antioxidant, anti-inflammatory, antimicrobial, and hypoglycemic properties [18,19]. *G. resinaceum* extracts have shown inhibitory effects against enzymes such as acetylcholinesterase, tyrosinase, α-amylase, and α-glucosidase [10,20]. Also, nortriterpenoids extracted from *Ganoderma* species display a diverse range of biological activities, such as antitumor, anti-inflammatory, neurotrophic, hepatoprotective, and anti-HIV-1 protease effects [8]. These properties are noteworthy due to their structural diversity and potential as models in pharmaceutical research. Additionally, polysaccharides such as β-glucans and phenolic compounds play significant roles, exhibiting anti-inflammatory, antimicrobial, antioxidant, and antiproliferative properties [6,7,10,11,12,20,21,22,23], while ergosterol peroxide has been shown to induce tumor cell death [24]. Wang et al. [9] proposed that the bioactive compounds from *G. pfeifferi* and other *Ganoderma* species may exert a synergistic effect, indicating that their medicinal properties typically result from the combination of multiple ingredients.

The genus is widely distributed across the globe, particularly in tropical and subtropical areas spanning Africa, America, Asia, and Europe [25]. In Europe, the genus consists of seven species [14], with *G. pfeifferi* and *G. resinaceum* being among the less studied species compared to *G. lucidum*, *G. applanatum*, and *G. adspersum*, among others.

Regarding taxonomy of these species, Rašeta et al. [10] claimed that *G. pfeifferi* and *G. resinaceum*, along with *G. subamboinense* and three *G. lucidum* strains from the United States and Taiwan, form a single monophyletic cluster based on phylogenetic analysis. This group is distinguished by its ability to produce chlamydospores in culture. Two selected species, *G. pfeifferi* and *G. resinaceum,* are from genus *Ganoderma*, which is the major genus of the *Ganodermataceae* family (Polyporales, Basidiomycota), known for producing bioactive compounds such as triterpenoids, polysaccharides, sterols, and phenolics [7,14].

Studies have also highlighted the efficacy of *Ganoderma* extracts in improving insulin sensitivity, regulating lipid metabolism, and reducing inflammation, as key factors in the management of metabolic syndrome [26,27,28,29]. The bioactive compounds in *Ganoderma* species can modulate multiple metabolic pathways in the body [30], making *Ganoderma* a promising candidate for the prevention and treatment of various chronic diseases [31].

As of today, the focus on antioxidant, antimicrobial, and enzyme inhibition, including in vitro antidiabetic activity [10,12,13,20,21,22,23,32,33], has focused on the new medicinal species, *G. pfeifferi*, originating from Serbia but also presents as an autochthonous European species, and *G. resinaceum*, distributed in Asia and North Africa as well.

In our previous study, we presented findings on the use of ethanolic (EtOH) and hot-water (H_2_O) extracts in suspension form derived from *G. pfeifferi* and *G. resinaceum*, which showed potential for application in diabetes [12]. Hence, the objective of this study was to conduct a thorough comparative assessment of the in vitro antiproliferative and in vivo lipid-lowering, and hepato- and nephroprotective characteristics of *G. pfeifferi* and *G. resinaceum*, an area notably deficient in current literature.

## 2. Materials and Methods

### 2.1. Fungal Material and Extracts Preparation

Fruiting bodies of two *Ganoderma* fungal species, namely *G. pfeifferi* and *G. resinaceum*, were gathered in September 2010 from the Nature Park Begečka Jama and the University of Novi Sad Campus in Serbia, respectively. The determination and identification of the collected specimens took place at the Department of Biology and Ecology, University of Novi Sad, and voucher specimens have been archived in the ProFungi Laboratory, Department of Biology and Ecology, University of Novi Sad (Serbia), under the numbers 12-00723 and 12-00722.

All experiments utilized EtOH and H_2_O extracts, prepared according to previously established methods [11,12]. Thirty grams of dried basidiomycete samples was crushed with 300 mL of 95% ethanol using a rotary shaker (IKA KS 3000i control, Wilmington, NC, USA) at 120 rpm for 72 h to obtain ethanol extracts. For hot-water extracts, maceration was carried out using boiled water, followed by incubation at 80 °C for 60 min in a water bath (Elektromedicine, Ljubljana, Slovenia). The resulting filtrates from the EtOH extracts were evaporated to dryness using a rotary evaporator (Büchi R-210; Büchi Labortechnik AG, Flawil, Switzerland) at 35 °C, while the H_2_O extracts were freeze-dried (Christ Alpha 1-2 LD Freeze Dryer, Osterode am Harz, Germany) for 72–96 h at an ice condenser temperature of −55 °C. Both extracts were dissolved in distilled water (dH_2_O), achieving a final concentration of 100 mg/mL dry weight (d.w.), and were stored at temperatures of −20 °C before undergoing analysis.

### 2.2. Mycochemical Characterization

#### 2.2.1. Quantification of Macro- and Microelements by Using Atomic Absorption Spectrophotometry (AAS)

Macro- (Ca and Mg) and microelements (Cu, Ni, Cd, Pb, Cr, Mn, Fe, and Zn) were analyzed in powdered fungal samples following the procedure outlined by Kebert et al. [34], using a flame technique within Atomic Absorption Spectrophotometry (AAS) (model FS AAS240/GTA120, Agilent) (Santa Clara, CA, USA). Approximately 0.3 g of dried material (oven-dried at 70 °C for 24 h) was ground, homogenized, and digested in a mixture of 10 mL of nitric acid and 2 mL of 30% (*w*/*v*) hydrogen peroxide using a microwave-assisted digestion system (D series; Milestone, Bergamo, Italy) at 180 °C (900 W) for 45 min. The resulting homogenates were filtered and diluted to 25 mL with deionized water. The prepared samples were analyzed by using the acetylene/air burner flame technique for Cr, Cu, Mn, Fe, and Zn and the nitrous oxide (N_2_O)–acetylene flame for Ca content determination. Concentrations of each element were determined using single-element hollow-cathode lamps at specific wavelengths and expressed as mg/kg dry weight (d.w.) of fungal material.

#### 2.2.2. LC-MS/MS Analysis of Phenolic Compounds

LC-MS/MS analysis involved quantifying quinic acid and seven other phenolic compounds using a previously established method [35]. The analysis utilized an Agilent Technologies 1200 Series high-performance liquid chromatograph coupled with an Agilent Technologies 6410A Triple Quad tandem mass spectrometer equipped with an electrospray ion source (Santa Clara, CA, USA). The system was controlled by Agilent Technologies MassHunter Workstation software—Data Acquisition (ver. B.06.00). Samples and standards were prepared in 50% aqueous methanol (MeOH) at a concentration of 2 mg/mL. Injection of a 5 μL sample into a Zorbax Eclipse XDB-C18 column (50 mm × 4.6 mm, 1.8 μm) achieved separation. Data were acquired in the dynamic Multiple Reaction Monitoring (MRM) mode and analyzed for peak areas using Agilent MassHunter Workstation Software—Qualitative Analysis (ver. B.06.00). The concentrations of the compounds in the extracts were determined by generating calibration curves using OriginLabs Origin Pro (ver. 2019) software.

#### 2.2.3. Total Carbohydrate Content (TCC)

The total carbohydrate content (TCC) of the fungal extracts was determined using the phenol–sulfuric acid method outlined in Rašeta et al. [12]. Initially, 50 μL of each fungal extract or glucose was mixed with 150 μL of concentrated H_2_SO_4_ (or dH_2_O for correction) and shaken for 30 min at room temperature in an incubator shaker (IKA KS 4000i control, Staufen, Baden-Württemberg, Germany). Subsequently, 30 μL of 5% phenol in water was added, and the mixture was heated for 10 min at 70 °C in the same incubator shaker. After allowing the microplate (Thermo Fisher Scientific, Waltham, MA, USA) to cool to room temperature for 5 min in a water bath, it was dried, and absorbances (Thermo Fisher Scientific, Waltham, MA, USA) were taken at 490 nm for glucose (hexose), 750 nm for sucrose as a disaccharide, and 480 nm for xylose (pentose). Calibration curves using glucose, sucrose, and xylose (13.59–2173.91 μg/mL) were prepared to determine the total carbohydrate content of the fungal extracts, expressed as mg glucose equivalents (mg GluE), mg sucrose equivalents (mg SucE), and mg xylose equivalents (mg XylE), per gram of dry weight (d.w.).

### 2.3. In Vitro Examination of Biological Activities

#### 2.3.1. Antioxidant Activity

The antioxidant activity was assessed through the ABTS radical scavenging activity assay [36] and OH radical scavenging activity assay [37], while the reducing power of the fungal extracts was determined using the ascorbate equivalent antioxidant capacity (A.E.A.C.) assay [38]. The scavenging activity in the case of the ABTS assay and the reducing power of the extracts were determined based on the standard curve equation of Trolox and ascorbic acid, respectively, while the results of the scavenging OH assay were expressed as IC_50_ values (concentration that inhibits 50% of hydroxyl radicals). The results were quantified as milligrams of Trolox equivalents per gram of dry weight (mg TE/g d.w.) and milligrams of ascorbic acid equivalents per gram of dry weight (mg AAE/g d.w.).

#### 2.3.2. Antiproliferative Activity

The antiproliferative activity of the analyzed fungal extracts was assessed using the estrogen-dependent breast cancer cell line (MCF-7), following the method outlined by Mosmann [39]. Ellagic acid was utilized as a positive control agent. Cancer cell viability was monitored over a 24-h (acute) and 72-h (chronic) incubation period for extract concentrations ranging from 50 to 250 µg/mL. Cell cytotoxicity was determined as IC_50_, which represents the concentration that inhibits 50% of cell growth extrapolated from concentration–response curves.

### 2.4. In Vivo Procedures and Assays

#### 2.4.1. Laboratory Animals

The in vivo part of the research was conducted on white Wistar rats of both genders, obtained from the Military Medical Academy of Belgrade, Republic of Serbia. The rats, weighing between 210 and 340 g and aged up to four months, were accommodated in UniProtect airflow cabinets (Ehret GmbH, Emmendingen, Germany) with standard plexiglass cages. The housing conditions maintained a constant room temperature of 22 ± 1 °C, 55% ± 1.5% humidity, and a regular circadian rhythm (12-h day/night cycle). Throughout the entire experiment, the rats were provided with unrestricted access to tap water and standard pelleted laboratory rodent feed from the Veterinary Institute Subotica, Serbia. All experimental procedures adhered to the guidelines of the European Directive (2010/63/EU) for animal experiments and were subject to review and approval by the Ethics Committee for the Protection and Welfare of Experimental Animals at the University of Novi Sad, Serbia.

#### 2.4.2. Experimental Procedures

The animals were randomly allocated into ten groups, each comprising six individuals. Among these, five groups underwent no alloxan pre-treatment (normoglycemic), while the remaining five groups were subjected to alloxan-induced hyperglycemia (diabetic). In order to induce diabetes, alloxan was dissolved in saline and applied intraperitoneally at a dose of 100 mg/kg. Hyperglycemia was confirmed 48 h following the application of alloxan, and animals with a glycemia higher than 15 mmol/L were selected for subsequent experiments. Both normoglycemic and diabetic control groups of animals were treated with saline at a dose of 1 mg/mL. Similarly, experimental groups of normoglycemic and diabetic animals were subjected to identical treatment, receiving an oral aqueous suspension (1 mg/mL) containing the EtOH and H_2_O extracts of the two analyzed fungal species. The extract suspensions were administered per orally by a nasogastric probe over a 5-day period. Two hours after the last dose of the fungal extracts or saline, the rats were anesthetized using a 25% solution of urethane at a dose of 5 mL/kg via intraperitoneal injection. Once the righting reflex was lost, the animals were exsanguinated through intracardial puncture to obtain blood and tissue samples for further analysis.

#### 2.4.3. In Vivo Biochemical Parameters Analysis

The concentration of lipids (lipid status) was determined in the serum of animals. The concentrations of total cholesterol, total triglycerides, and high-density lipoprotein (HDL) cholesterol and low-density lipoprotein (LDL) were measured using clinical biochemistry methods.

The enzymatic activity of aspartate aminotransferase (AST) and alanine aminotransferase (ALT), as well as the concentrations of urea and creatinine, were assessed in order to monitor hepatic and renal function using serum of the examined animals.

All analyses were performed using standard spectrophotometric methods on an automatic chemical analysis system, Olympus AU 400 (Hamburg, Germany).

### 2.5. Statistical Analysis

The study utilized an array of statistical methods, including descriptive statistics, one-way Analysis of Variance (ANOVA), *t*-tests, Principal Component Analysis (PCA), dendrogram hierarchical clustering, and Pearson correlation analysis. The differentiation among analyzed fungi species in the one-way ANOVA was assessed using the Fisher (F) test, with statistical significance denoted by “*p*-values”. The results of the *t*-tests were depicted with box-plot diagrams. All statistical analyses were performed using the R programming environment. The “rstatix” R package was employed for descriptive statistics, two-way ANOVA, and *t*-tests, while dendrogram clustering was carried out with the “dendextend” R package. Various data visualizations were created using the “ggplot2” R package [40,41,42].

## 3. Results and Discussion

### 3.1. Mycochemical Characterization

#### 3.1.1. AAS Quantification of Macro- and Microelements

**Figure 1:**
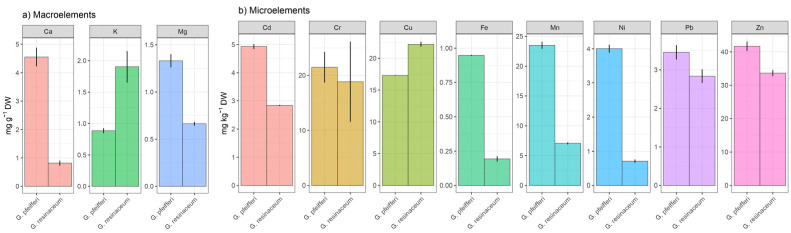
(**a**) Composition of macroelements (mg/g d.w.) and (**b**) microelements (mg/kg d.w.) in *G. pfeifferi* and *G. resinaceum* samples.

AAS analysis was utilized to evaluate the multi-elemental composition of both *G. pfeifferi* and *G. resinaceum*, encompassing a total of 11 metals. These included macroelements such as Ca, K, and Mg, and microelements like Cu, Ni, Cd, Pb, Cr, Mn, Fe, and Zn. The summarized results can be found in Figure 1a,b.

Figure 1a,b display the outcomes of the mineral composition analysis, highlighting the prevalence of three major macroelements, namely Mg, Ca, and K. In *G. pfeifferi*, the predominant macroelements were Mg and Ca, constituting a substantial portion of the overall mineral composition [33], which aligns with previous studies highlighting the essential role of Mg and Ca in various physiological processes, including the immune regulating actions of Mg and its crucial role in regulating inflammation and immune response to infectious agents and malignancies [43,44]. Contrastingly, *G. resinaceum* exhibited a distinctive macroelement profile, with a notable abundance of K. The significance of K in cellular activities, particularly in maintaining osmotic balance and regulating enzyme functions, suggests potential therapeutic implications associated with *G. resinaceum* consumption [45,46].

Beyond macroelements, the AAS analysis also unveiled the presence of essential microelements in both *Ganoderma* species (Figure 1b). It is noteworthy that elements such as Zn and Cu were detected in appreciable amounts in both tested species, with a higher concentration of Zn in *G. pfeifferi* (41.70 ± 1.11 mg/kg d.w.), in contrast to *G. resinaceum*, where Cu was a more dominant microelement (22.22 ± 0.48 mg/kg d.w.). Moreover, the concentration of Cu in *G. resinaceum* from Poland was two times lower (11.00 ± 3.00 mg/kg d.m.) [47] compared to the concentration determined in this study, while Zn was not quantified. These microelements play crucial roles in enzymatic activities, oxidative stress defense, and overall metabolic processes [48,49].

The comparative assessment of the mineral composition between *G. pfeifferi* and *G. resinaceum* unveiled species-specific variations and the prevalence of tested elements in *G. pfeifferi*. This aligns with the previous report where Marek et al. [47] suggested that among the tested group of *Ganoderma* species, fruit bodies of wild-growing *G. resinaceum* and cultivated *G. pfeifferi* were characterized by a higher level of all elements jointly than the other analyzed *Ganoderma* species. Rašeta et al. [50] highlighted that the mineral content of edible fungi after consumption is influenced by cooking or processing methods, often resulting in mineral leakage into water or brine. While these minerals offer nutritional benefits, excessive intake may pose risks, especially considering fungus’ ability to accumulate toxic elements and radionuclides. Therefore, it is crucial to assess mineral content before consuming wild fungi and consume them in moderation.

Considering this information and as depicted in Figure 1b, it can be inferred that *G. pfeifferi* and *G. resinaceum* exhibited negligibly higher accumulation of toxic elements such as the examined Cd and Pb (ranging from 2.84 to 4.93 mg/kg and 2.84 to 3.45 mg/kg d.w., respectively), compared to the other two *Ganoderma* species originating from Serbia as well (Cd ranging from 0.82 to 1.79 mg/kg and Pb ranging from 2.22 to 3.70 mg/kg for *G. applanatum* and *G. lucidum*, respectively) [11]. Such distinctions may influence the therapeutic potential of these fungi, as the interplay between different elements could contribute to their observed biological activities. Conversely, Gałgowska and Pietrzak-Fiećko [51] conducted a study on the Pb and Cd content in edible fungi—*Boletus badius*, *B. edulis*, and *Cantharellus cibarius*—from northeastern Poland, estimating their safety for human consumption. They found that safe concentrations of Pb and Cd in fungi should be below 0.424 mg/kg d.w. for Pb and below 2.151 mg/kg d.w. for Cd. However, comparing the accumulation of heavy metals between edible and medicinal fungal species, as studied here, is challenging due to their distinct methods of consumption. Singh and Nyau [52] affirmed this, highlighting that different fungal species exhibit varying biosorption efficiencies for specific heavy metals. This indicates that edible and medicinal mushrooms likely accumulate heavy metals differently. Moreover, direct comparisons of heavy metal accumulation between these two types of fungi are limited because they are consumed differently: edible fungi are consumed directly, whereas medicinal fungi are typically used in processed forms for their therapeutic benefits.

In summary, the mineral composition analysis of *G. pfeifferi* and *G. resinaceum* indicated significant concentrations of biogenic metals alongside minor levels of heavy metals, potentially attributable to bioaccumulation processes, which is in accordance with Yalcin et al. [33]. Consequently, there arises a necessity for the regulated cultivation of these fungi with promising medicinal attributes. Moreover, understanding the mineral composition of *Ganoderma* species is pivotal for unraveling the potential therapeutic benefits associated with these fungal species.

#### 3.1.2. Total Carbohydrate Content (TCC)

**Figure 2:**
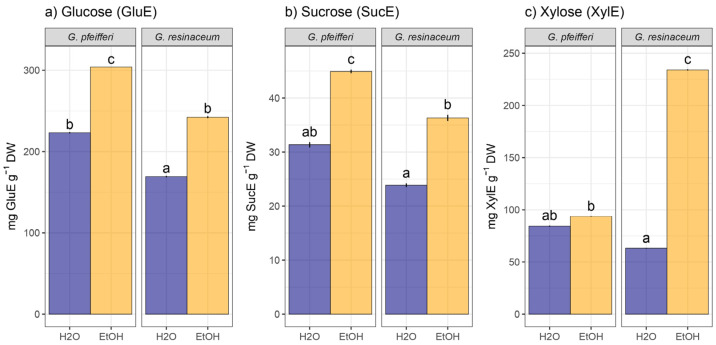
Total carbohydrate content: (**a**) glucose, (**b**) sucrose, and (**c**) xylose. Different small letters indicate significant differences among different analyzed extracts of *G. pfeifferi* and *G. resinaceum*; Tukey’s significant difference (HSD) post hoc test (*p* ≤ 0.05). Data represent the mean ± standard deviation (SD).

Regarding the TCC analysis, it is important to highlight that the EtOH extracts of *G. pfeifferi* demonstrated a notably higher concentration of glucose and sucrose (303.87 ± 54.80 mg GluE/g d.w. and 44.51 ± 9.49 mg SucE/g d.w., respectively) compared to the same extract of *G. resinaceum*, indicating a substantial disparity in their carbohydrate composition (Figure 2). Conversely, *G. resinaceum* EtOH exhibited increased xylose content (233.18 ± 11.37 mg XylE/g d.w.), whereas all hot-water extracts displayed statistically significant lower TCC in both species (Figure 2).

These findings align with our earlier investigation, wherein EtOH extracts demonstrated superiority in TCC compared to water extracts, accompanied by a higher TCC content measured in glucose equivalents in *G. pfeifferi* [10]. On the other hand, higher TCC was determined in the study of *G. resinaceum* from Serbia, where 52.1 ± 3.2 g GluE/100 g was quantified in the hot-water extract [32]. This discrepancy in carbohydrate levels between the two species and the type of solvent used suggests that the extraction method, together with geographical origin, i.e., ecological factors, may have implications for their nutritional value and potential applications, as suggested for other fungal species [53]. Quantification of monosaccharide content in the analyzed species represents a valuable report, since, generally, fungal polysaccharides are composed of glucose, galactose, and mannose, but other carbohydrates can also be found (e.g., xylose, arabinose, fucose, ribose) [54], as demonstrated in this study as well. Since the ratio of monosaccharide composition in fungi is very important and it has been shown that polysaccharides from *G. lucidum* have positive effects as hypoglycemic agents [55], future research should be based on detailed identification of polysaccharides, including monosaccharide composition, from the analyzed two *Ganoderma* species as well.

### 3.2. Biological Activities of the Examined Extracts

#### 3.2.1. Antioxidant Activity

**Figure 3:**
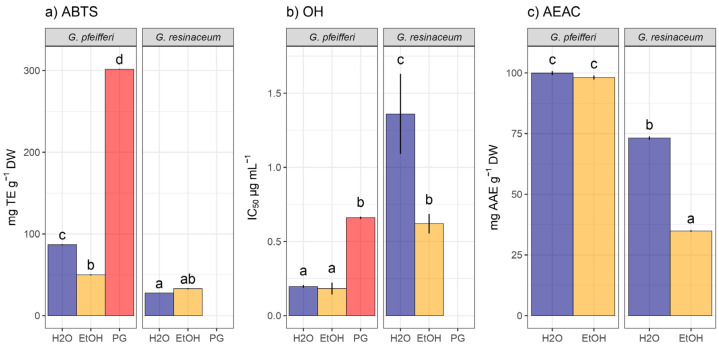
Antioxidant activity in hot-water (H_2_O) and ethanolic (EtOH) extracts of *G. pfeifferi* and *G. resinaceum*: (**a**) RSC against ABTS radical (2,2′-azinobis-3-ethylbenzothiazoline-6-sulfonic acid); (**b**) radical scavenger capacity against OH radical; (**c**) ascorbate equivalent antioxidant capacity (A.E.A.C.) assay. The distinct lowercase letters denote significant differences observed among different analyzed samples, including the standard compound propyl gallate (PG), as determined by Tukey’s honestly significant difference (HSD) post hoc test (*p* ≤ 0.05). The data are presented as the mean ± standard deviation (SD).

The antioxidant activity of *Ganoderma* species was assessed through ABTS, OH, and A.E.A.C assays, revealing varying degrees of efficacy across the tested species (Figure 3).

Results from the ABTS assay indicated potent antioxidant capacity in the *G. pfeifferi* H_2_O extracts (86.85 ± 0.67 mg TE/g d.w.), while *G. resinaceum* exhibited a comparatively lower neutralization of ABTS radical. Furthermore, there was no statistically significant distinction observed in the neutralization of this radical between the EtOH and H_2_O extracts of *G. resinaceum*. This similarity is also evident in the extracts of *G. pfeifferi*, albeit in the neutralization of the OH radical, where extracts demonstrated notable antioxidant potential in comparison with the analyzed standard compound (PG) (Figure 3). Interestingly, the A.E.A.C. assay also highlighted *G. pfeifferi* as possessing the highest reduction power activity, compared to the reduction ability of *G. resinaceum* extracts.

In samples of *G. resinaceum* obtained from Turkey as well, Zengin et al. [20] determined lower antioxidant potential in comparison with the results of this study, underscoring the diverse antioxidant properties among *Ganoderma* species. Results from this study are in accordance with the study of Yalcin et al. [33], where extracts of *G. pfeifferi* from Turkey showed higher reduction potential compared to neutralization of ABTS radicals. Moreover, compared to our results, the H_2_O extracts of *G. pfeifferi* exhibited a two-times higher ability of ABTS radical neutralization (170.32 ± 3.17 mg TE/g) [33]. Recently, Sułkowska-Ziaja et al. [13] conducted antioxidant analyses on both studied species. In comparison with our findings, methanolic extracts from mycelial cultures (Lublin, Poland) exhibited lower activity in the ABTS assay (9.77 ± 0.13 mg TE/g for *G. pfeifferi* and 11.60 ± 0.36 mg TE/g for *G. resinaceum*, respectively), while the reduction potential of the samples analyzed by Sułkowska-Ziaja et al. [13] was also lower (ranging from 8.10 to 31.78 mg TE/g). This suggests that the choice of extract solvent and preparation method could influence experimental outcomes.

**Figure 4:**
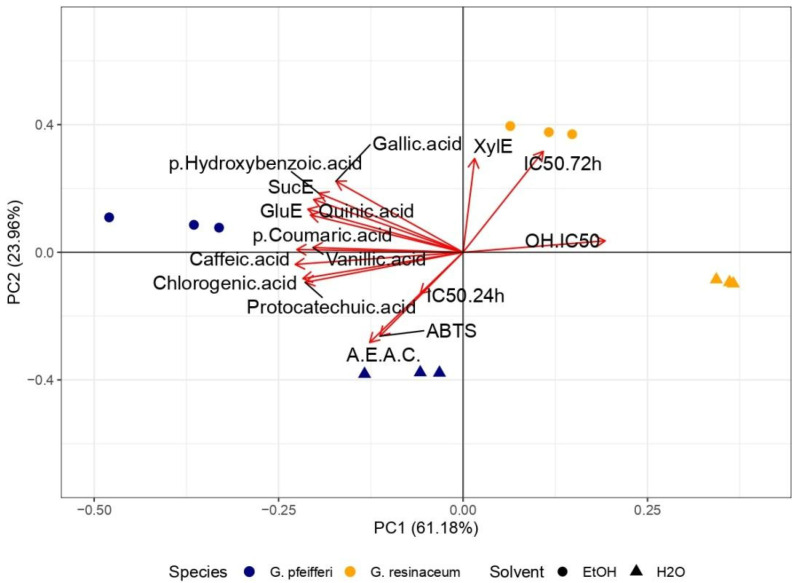
Principal Component Analysis (PCA) of the observed antioxidant and antiproliferative activity along with phenolic compounds (Table S1) [12] and total carbohydrate content (TCC) in the four examined fungal extracts of *G. pfeifferi* and *G. resinaceum*. The following are the abbreviations of the examined parameters: EtOH—ethanolic extract; H_2_O—hot-water extract; ABTS—2,2′-azinobis(3-ethylbenzothiozoline)-6-sulfonic acid; OH—hydroxyl radical; A.E.A.C.—ascorbate equivalent antioxidant capacity; IC_50_ 24 h/72 h—antiproliferative inhibitory concentration in the case of incubation of 24 h and 72 h; GluE—glucose equivalents; SucE—sucrose equivalents; XylE—xylose equivalents; Tukey’s honestly significant difference (HSD) post hoc test (*p* ≤ 0.05). Data represent the mean ± standard deviation (SD).

PCA analysis was conducted to reveal a connection between the antioxidant activity and the detected TCC, alongside our previous research (Table S1) [12], where the phenolic profile was determined in the tested extracts. PCA analysis revealed a 60.40% variance of PC1 and 25.97% of PC2, while distinct clustering may be observed among the two analyzed *Ganoderma* species (Figure 4).

**Figure 5:**
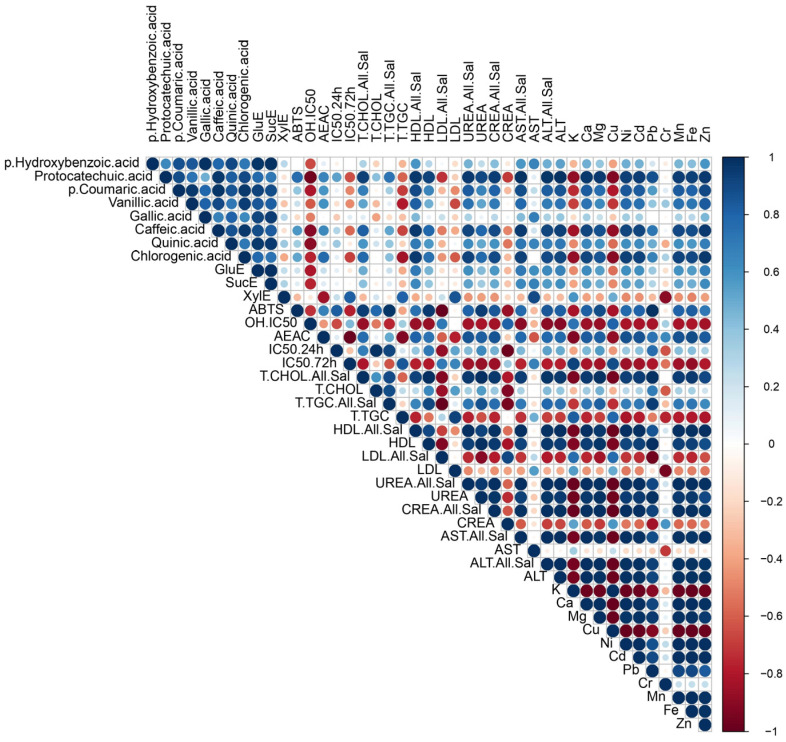
Pearson’s coefficient of the correlation matrix of the examined parameters in the ethanolic (EtOH) and hot-water (H_2_O) extracts of *G. pfeifferi* and *G. resinaceum* collected on the territory of the Republic of Serbia. Blue squares represent a highly significant correlation of inspected parameters, while red squares present low interactions, assessed according to the corresponding Pearson’s coefficient. The following are the abbreviations of the examined parameters: ABTS—radical scavenger capacity against 2,2′-azinobis(3-ethylbenzothiozoline)-6-sulfonic acid, ABTS^•+^; OH—radical scavenger capacity against hydoxyl radical, OH^•^; A.E.A.C.—ascorbate equivalent antioxidant capacity; IC_50_ 24 h/72 h—antiproliferative inhibitory concentration in the case of incubation of 24 h and 72 h; GluE—glucose equivalents content of TCC; SucE—sucrose equivalents content of TCC; XylE—xylose equivalents content of TCC; All.Sal—alloxan + saline; CHOL—cholesterol; T.TGC—total triglycerides; CREA—creatinine; ALT—alanine aminotransferase and AST—aspartate aminotransferase.

Evidently, *G. resinaceum* separated in the positive quadrant of both PCs, opposite to the *G. pfeifferi* extracts and all quantified compounds, suggesting a negative correlation among phenolics (Table S1), TCC, and antioxidant properties of the *G. resinaceum* extracts [12] (Figure 4). On the contrary, in the *G. pfeifferi,* extracts clustered together with TCC and all identified phenolics, suggesting their role as strong antioxidative agents in this species. Moreover, this is in accordance with correlation analysis where a strong positive correlation among antioxidant properties, except in the case of the neutralization of OH radical and phenolic compounds, is evident (Figure 5). Also, this assumption was confirmed in previous research as well [10,11,12,20,21,53]. However, the separation of the *G. resinaceum* extracts in the PCA graph, opposite to phenolics (Table S1), could be related to lower levels of these compounds and, thus, the lower antioxidant properties observed.

#### 3.2.2. Antiproliferative Activity

The MTT assay was utilized to assess the in vitro antiproliferative activity of the two analyzed crude extracts and standard compound (ellagic acid). According to data from Table 1, it is evident that all extracts demonstrated antiproliferative effects during subacute incubation (after 24 h), with the most potent activity observed in the H_2_O extract of *G. resinaceum* species (IC_50_ = 4.88 ± 0.50 μg/mL), comparable to the activity of the standard compound (IC_50_ = 33.94 ± 3.69 μg/mL for ellagic acid). In general, the EtOH extracts showed less inhibition of MCF-7 cells, except for the *G. pfeifferi* EtOH extract, which exhibited significant subacute inhibition (IC_50_ = 154.05 ± 12.92 μg/mL).

PCA analysis was performed to establish a link between the antiproliferative activities observed in this study and detected TCC, together with our prior research findings (Table S1) [12], where the phenolic profile was determined in the tested extracts. The PCA analysis demonstrated distinct clustering patterns among the *Ganoderma* species based on their antiproliferative activity. The initial pair of principal components, PC1 and PC2, explained 86.37% of the overall variance, demonstrating a significant portrayal of the dataset. Specifically, *G. resinaceum,* which exhibited the highest antiproliferative activity, separated in the positive quadrant of both PCs, opposite to *G. pfeifferi* and all quantified compounds [12] (Figure 4). This is in accordance with the obtained results since lower levels of phenolic compounds (Table S1) and TCC were observed in the *G. resinaceum* EtOH and H_2_O extracts. On the other hand, the correlation matrix revealed a positive correlation between xylose and antiproliferative activity (Figure 5), suggesting that maybe carbohydrate compounds are important for cytotoxic effect. In contrast, extracts from *G. pfeifferi* exhibited diminished antiproliferative activity yet demonstrated elevated levels of TCC (Figure 4) and phenolic content, as presented in Table S1, clustering together within the I and III quadrants. Moreover, the correlation matrix showed that only protocatechuic acid, one of the most abundant phenolics in the *G. pfeifferi* extracts (Table S1), had a significant positive correlation with antiproliferative activity (Figure 5).

Rašeta et al. [11] summarized that extensive research conducted in recent decades has identified a broad spectrum of bioactive compounds extracted from *Ganoderma* species, including phenolic acids, isoflavones, polysaccharides, triterpenes, sterols, nucleosides, proteins, and polysaccharide-protein complexes, all with potential antiproliferative effects. Considering the analysis of the MCF-7 cell line with unpurified extracts, it is hypothesized that interactions among various biomolecules present in the tested extracts may synergistically demonstrate antiproliferative activity, potentially mitigating the toxicity of individual components. This suggests that the interaction between different biomolecules could enhance the therapeutic efficacy of the *Ganoderma* crude extracts [11]. However, drawing on previously reported data, it is theorized that the polysaccharides present in the analyzed hot-water extracts are responsible for the antiproliferative activity, while terpenoids potentially present in the EtOH extracts contribute primarily to a proliferative effect on MCF-7 cells [11]. Consequently, it can be inferred that this effect is linked to the direct antiproliferative treatment against tumor cells [56]. The same group claimed that high-molecular-weight fungal compounds such as polysaccharides and polysaccharide–protein complexes are significant for the exhibition of antitumor activity due to their increased solubility in water [56]. The most active polysaccharides of *Ganoderma* belong to β-(1-3)-D-glucans, well known for the promotion of antitumor activity in animals and humans by acting as immune modulators—biological response modifiers, because they promote natural and acquired immunity of the host organism itself [11].

In contrast, our results indicated that the identified phenolic compounds (Table S1) and TCC were probably not responsible for the detected antiproliferative activity, especially in *G. resinaceum*. This suggests that terpenoid compounds could play the main role in this activity [19] since various extracts from *Ganoderma lucidum* enriched with triterpenoids inhibit the growth of hepatoma cells by suppressing protein kinase C and activating mitogen-activated protein kinases [57]. Also, various types of ganoderic acids, including ganoderic acid T and its C-3 epimer compound, isolated from *G. orbiforme,* showed a cytotoxic effect on MCF-7 cells [58], indicating that in this study, triterpenoids could be responsible for the high antiproliferative activity of *G. resinaceum*, as well. The results of the study by Rikame et al. [59] support this assumption since terpenoids were quantified as major components in the *G. resinaceum* extracts and exhibited cytotoxic activity against human colon HCT 116 cancer cells, while ganoresinoid A from this species alleviated LPS-induced apoptosis, as described by Kou et al. [60], but further research should be conducted.

#### 3.2.3. Lipid-Lowering Properties

Diabetes mellitus was induced in the animals, leading to dyslipidemia characteristic of this disease, as best observed in the group receiving a physiological solution for five days after diabetes induction. In this group and based on Table 2, a drastic increase in serum total triglyceride concentration (4.19 mmol/L) was evident, which was significantly higher compared to the total triglyceride values of the control group (1.14 mmol/L) and all other experimental groups treated with the fungal extracts. Another laboratory parameter supporting diabetic dyslipidemia was the elevated level of LDL cholesterol fraction, significantly higher in the group of animals with diabetes treated with the physiological solution compared to all other investigated groups.

The animal model of alloxan-induced diabetes effectively mirrors lipid metabolism disturbances in humans with hyperglycemia. This primarily leads to an increase in blood triglycerides as an alternative energy source, resulting in the elevation of very-low-density lipoproteins (VLDL) and, subsequently, the highly atherogenic LDL fraction. Therefore, considering the clinical therapeutic goal, the focus is primarily on lowering LDL cholesterol and secondarily on total triglycerides [61].

The valuable nutritional profile and benefits of using fungi in hyperglycemic conditions have been demonstrated in our previous studies [12,62,63]. In this study, it is evident that treatment with extracts from both fungal species of the genus *Ganoderma* prevented the disturbance in lipid status common in diabetes mellitus. There is even a tendency toward an increase in HDL cholesterol in certain groups of diabetic animals treated with fungal extracts, although unfortunately, statistical significance was not reached.

Furthermore, clinical studies have indicated a connection between dyslipidemia and the risk of cancer development, emphasizing that anything favorably impacting the lipid profile can be beneficial in prevention. In addition to showing antiproliferative potential, the examined *Ganoderma* species demonstrated a positive effect on the in vivo lipid status, representing their additional pharmacological value [64,65].

In addition to lipid metabolism disturbances in diabetes, changes in renal and hepatic tissues also occur, as observed in this study. Serum levels of urea and creatinine, biochemical indicators of kidney function, were significantly elevated in the group of diabetic animals treated with physiological solution compared to the control group of healthy animals (urea—46.98:7.40 mmol/L; creatinine—289.25:52.25 mmol/L). In all normoglycemic animals treated with fungal extracts, the serum concentration of urea and creatinine remained at the level of the control group values, which was not the case for diabetic animals. Neither the H_2_O nor EtOH extract of *G. pfeifferi* succeeded in preventing the increase in kidney function parameters; however, both extracts of *G. resinaceum* achieved this. Although it has been demonstrated that meroterpenoids from fungi of the genus *Ganoderma* exhibit renoprotective effects [66,67], we consider that the treatment duration with the extracts in this study was too short for a direct impact on renal tissue. Nevertheless, it is possible that indirectly, through the regulation of glycemia, as shown in our previous research [12], there was a positive influence on serum levels of urea and creatinine in the case of *G. resinaceum*.

Regarding liver function, the ANOVA test indicated no statistically significant differences in AST values when comparing all groups of animals. In contrast to AST, the values of ALT, which is a more specific indicator of liver function, differ significantly. It was observed that serum ALT values were lower in all groups of animals treated with the extract of *G. resinaceum* compared to all *G. pfeifferi* groups, as well as the control groups of normoglycemic and diabetic animals. Additionally, the ALT concentration in groups of diabetic animals treated with a physiological solution or the *G. pfeifferi* extracts was significantly lower compared to the control normoglycemic group. However, it cannot be confidently claimed that a hepatoprotective effect has been demonstrated by only considering the result that compared to normoglycemic control in most groups of animals treated with fungal extracts, a decrease in ALT values was evident. In our previous study, using the same model and experimental design, we showed that there were no noticeable histo-morphological changes in liver tissue indicating damage. The same study described that treatment with *G. pfeifferi* and *G. resinaceum* fungal extracts has a positive effect on liver biochemical parameters, with lipid peroxidation in the first place [12].

**Figure 6:**
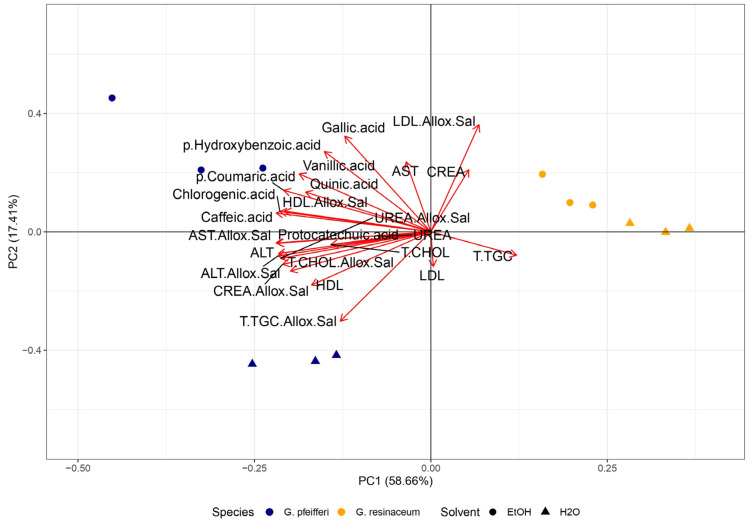
Principal Component Analysis (PCA) of the lipid-lowering properties along with phenolic compounds (Table S1) [12] and total carbohydrate content (TCC) in the four examined fungal extracts of *G. pfeifferi* and *G. resinaceum*. The following are the abbreviations of the examined parameters: EtOH—ethanolic extract; H_2_O—hot-water extract; Allox.Sal—alloxan + saline; CHOL—cholesterol; T.TGC—total triglycerides; CREA—creatinine; ALT—alanine aminotransferase and AST—aspartate aminotransferase; Tukey’s honestly significant difference (HSD) post hoc test (*p* ≤ 0.05). Data represent the mean ± standard deviation (SD).

PCA analysis and a correlation matrix for antidiabetic activity were also conducted to determine the correlation levels among this activity and determined bioactive compounds. PCA total variance was (76.07%), where PC1 was 58.66%, and PC2 was 17.41% (Figure 6).

Regarding PCA analysis, a similar pattern emerged as seen with antioxidant and antiproliferative activity (Figure 6), where extracts from *G. resinaceum* distinctly separated from those of *G. pfeifferi*, along with all previously identified phenolics (Table S1) and TCC. This implies that the potent antidiabetic activity observed in the *G. resinaceum* extracts cannot be solely attributed to phenolics and carbohydrates, suggesting that other compounds may likely play a pivotal role. To corroborate this, findings from other authors illustrate the hepatoprotective effect of terpenoid compounds isolated from *Ganoderma* fungi, such as ganomycin, fornicatin A, D, and F [9,67]. However, the role of phenolic compounds in antidiabetic activity should not be ignored since the correlation matrix revealed that the detected phenolic compounds (Table S1) showed a significant positive correlation with measured parameters regarding lipid-lowering properties of the examined extracts. This was also in accordance with our previous study [12] and Prabhakar [68], suggesting the necessity of further research and investigation of synergistic effects.

## 4. Conclusions

By comprehending the unique chemical compositions and therapeutic potentials of *G. pfeifferi* and *G. resinaceum*, these species could offer opportunities for the creation of innovative biopharmaceutical treatments, particularly for conditions that encompass both irregular cell growth and irregular lipid metabolism. The AAS quantification of micro and macroelements in *G. pfeifferi* and *G. resinaceum* provided valuable insights into the nutritional and therapeutic aspects of these medicinal fungal species. Further research exploring the biological activities associated with specific mineral compositions is warranted, as it could enhance our understanding of the potential health benefits conferred by these fungi.

Our findings underscored the therapeutic potential of *Ganoderma* extracts in ameliorating dyslipidemia associated with diabetes mellitus. Moreover, these extracts did not lead to organ damage; rather, they resulted in an improvement or no change in the biochemical parameters indicative of liver and kidney function compared to the control group. Further investigation into the underlying mechanisms and long-term effects of *Ganoderma* extracts on lipid metabolism and organ function is warranted for comprehensive understanding and clinical translation.

## Figures and Tables

**Table 1:** The antiproliferative effects of *Ganoderma* extracts and standard compounds on the MCF-7 cell line the MTT assay, IC_50_ values (μg/mL).

Incubation Period	Analyzed Samples				
	*G. pfeifferi* EtOH	*G. pfeifferi* H_2_O	*G. resinaceum* EtOH	*G. resinaceum* H_2_O	Ellagic Acid
24 h	154.05 ± 12.92 ^c^	653.35 ± 10.19 ^e^	363.87 ± 1.51 ^d^	4.88 ± 0.50 ^a^	33.94 ± 3.69 ^b^
72 h	78.33 ± 1.89 ^b^	49.25 ± 1.72 ^a^	181.07 ± 0.21 ^d^	113.33 ± 0.62 ^c^	43.06 ± 1.22 ^a^

IC_50_—extract concentration required to inhibit cell growth by 50%. Values are expressed as mean ± SD of triplicates. ^a–e^—different letters in the same row in comparison with ellagic acid as a standard compound indicate a significant difference between extracts (ANOVA, Tukey post hoc, *p* < 0.05).

**Table 2:** In vivo biochemistry parameters. The concentration of total cholesterol (mmol/L), triglycerides (TGC; mmol/L), HDL (mmol/L), and LDL (mmol/L) cholesterol, urea (mmol/L), and creatinine (mmol/L), as well as the enzymatic activity of aspartate aminotransferase (AST; U/L) and alanine aminotransferase (ALT; U/L) (mean value ± SD) in the serum of normoglycemic and diabetic (alloxan) rats treated with saline, EtOH and H_2_O extracts of the species *G. pfeifferi* and *G. resinaceum*.

	Parameter	Control	Alloxan + Saline	Alloxan + Saline + G.p. EtOH	G.p. EtOH	Alloxan + Saline + G.p. H_2_O	G.p. H_2_O	Alloxan + Saline + G.r. EtOH	G.r. EtOH	Alloxan + Saline + G.r. H_2_O	G.r. H_2_O
Lipid status	Total cholesterol	1.54 ± 0.24 ^a^	1.73 ± 0.24 ^a^	1.78 ± 0.25 ^a^	1.56 ± 0.34 ^a^	1.90 ± 0.26 ^a^	1.71 ± 0.19 ^a^	1.55 ± 0.23 ^a^	1.63 ± 0.32 ^a^	1.49 ± 0.18 ^a^	1.55 ± 0.21 ^a^
	Total TGC	1.14 ± 0.60 ^a^	4.19 ± 0.94 ^b^	1.09 ± 0.20 ^a^	0.89 ± 0.19 ^a^	1.71 ± 0.23 ^a^	1.090.40 ^a^	1.01 ± 0.30 ^a^	1.47 ± 1.09 ^a^	0.75 ± 0.21 ^a^	1.15 ± 0.51 ^a^
	HDL	0.90 ± 0.17 ^a^	0.75 ± 0.17 ^a^	1.01 ± 0.16 ^a^	0.90 ± 0.21 ^a^	0.97 ± 0.25 ^a^	0.98 ± 0.15 ^a^	0.79 ± 0.17 ^a^	0.78 ± 0.10 ^a^	0.77 ± 0.15 ^a^	0.74 ± 0.12 ^a^
	LDL	0.21 ± 0.10 ^a^	0.85 ± 0.40 ^b^	0.27 ± 0.14 ^a^	0.25 ± 0.12 ^a^	0.06 ± 0.06 ^a^	0.30 ± 0.13 ^a^	0.32 ± 0.12 ^a^	0.35 ± 0.10 ^a^	0.37 ± 0.16 ^a^	0.27 ± 0.15 ^a^
Renal function	Urea	7.40 ± 0.56 ^a^	46.98 ± 6.01 ^b^	76.64 ± 7.63 ^c^	8.50 ± 1.37 ^a^	74.32 ± 10.97 ^c^	8.73 ± 1.21 ^a^	6.23 ± 0.73 ^a^	8.02 ± 1.49 ^a^	6.27 ± 0.64 ^a^	8.00 ± 0.85 ^a^
	Creatinine	52.25 ± 0.62 ^a^	289.25 ± 73.87 ^b^	379.00 ± 79.09 ^c^	51.00 ± 3.03 ^a^	395.40 ± 97.39 ^c^	48.50 ± 2.43 ^a^	48.50 ± 4.72 ^a^	50.40 ± 9.02 ^a^	50.00 ± 6.45 ^a^	52.83 ± 8.47 ^a^
Liver function	AST	268.00 ± 44.62 ^a^	310.50 ± 33.29 ^a^	320.80 ± 41.41 ^a^	305.40 ± 66.41 ^a^	308.17 ± 52.32 ^a^	288.83 ± 44.59 ^a^	224.67 ± 70.21 ^a^	319.83 ± 10.76 ^a^	207.50 ± 38.87 ^a^	284.67 ± 18.33 ^a^
	ALT	98.00 ± 13.00 ^c^	60.17 ± 9.64 ^b^	50.17 ± 14.16 ^b^	83.60 ± 18.35 ^c^	51.83 ± 12.59 ^b^	82.17 ± 15.54 ^c^	23.20 ± 3.90 ^a^	34.60 ± 10.78 ^a^	17.833.25 ^a^	24.208.64 ^a^

Data represent the mean ± standard deviation (SD). ^a–c^ Results that do not share the same superscript in the same column are statistically significant. *p* < 0.01 (Tukey test, ANOVA).

## Data Availability

Data are contained within the article and supplementary materials.

## Supplementary Materials

The following supporting information can be downloaded at: https://www.mdpi.com/article/10.3390/jof10070501/s1, Table S1: LC-MS/MS detection of phenolic compounds in examined extracts.
