# Supplementary material for: Ganoderma pfeifferi Bres. and Ganoderma resinaceum Boud. as Potential Therapeutic Agents: A Comparative Study on Antiproliferative and Lipid-Lowering Properties
Source: J Fungi (Basel). 2024 Jul 19;10(7):501. doi: 10.3390/jof10070501 (PMC11277669; doi:10.3390/jof10070501)
Supplement: Supplementary file 1 [file jof-10-00501-s001.zip › jof-3088064-supplementary.pdf]

Journal of Fungi

MDPI

Table S1. LC-MS/MS detection of phenolic compounds in examined extracts[a]

Phenolic compund	Fungal species and extract type<br>Amount of compound detected (µg per g d.w.)			
	G. pfeifferi EtOH	*G. pfeifferi* H_2O	*G. resinaceum* EtOH	*G. resinaceum* H_2O
p-Hydroxybenzoic acid	23.00	5.10	12.20	<0.30b
Protocatechuic acid	**6.50**	**6.20**	**4.01**	**2.65**
p-Coumaric acid	**1.50**	**1.00**	**0.80**	**0.60**
Vanillic acid	**6.50**	**4.50**	<4.00[b]	<4.00[b]
Gallic acid	**30.50**	**1.50**	**15.85**	**1.20**
Caffeic acid	**0.80**	**0.60**	**0.40**	**0.25**
Quinic acid	**8.51**	**6.35**	**6.90**	**3.00**
Chlorogenic acid	**1.26**	**0.80**	<0.30[b]	<0.3[0b]

[a]EtOH, ethanolic extract; H_2O, water extract. Bold number: amount of quantified phenolic compounds in examined extracts. [b]Number: detected compound – peak observed, concentration is lower than the LoQ (limit of quantification), but higher than LoD (limit of detection).
